# A mixed-methods survey to explore issues with virtual consultations for musculoskeletal care during the COVID-19 pandemic

**DOI:** 10.1186/s12891-021-04113-y

**Published:** 2021-03-05

**Authors:** Anthony W Gilbert, Gregory Booth, Tony Betts, Andy Goldberg

**Affiliations:** 1 Therapies Department, Royal National Orthopaedic Hospital, Stanmore, UK; 2 School of Health Sciences, University of Southampton, Southampton, UK; 3 Institute of Orthopaedics and Musculoskeletal Sciences, University College London, London, UK; 4 Trauma and Orthopaedics Department, Wellington Hospital, London, UK; 5 MSK Lab, Imperial College London, London, UK

## Abstract

**Objective:**

To explore orthopaedic and musculoskeletal clinicians’ views and experiences of legal, safety, safeguarding and security issues regarding the use of virtual consultations (VC) during the COVID-19 pandemic. A secondary objective was to suggest ways to overcome these issues.

**Methods:**

A mixed method cross-sectional survey was conducted, seeking the views and experiences of orthopaedic and musculoskeletal medically qualified and Allied Health Professionals in the United Kingdom. Descriptive statistical analysis was employed for quantitative data and a qualitative content analysis undertaken for qualitative data. Findings were presented in accordance with the four key issues.

**Results:**

Two hundred and ninety professionals (206 physiotherapists, 78 medically qualified professionals, 6 ‘other’ therapists) participated in the survey. Of the 290 participants, 260 (90%) were not using VC prior to the COVID-19 pandemic, 248 respondents (86%) were unsure whether their professional indemnity insurance covered VC, 136 (47%) had considered how they would handle an issue of safeguarding whilst the remainder had not, 126 (43%) had considered what they would do if, during a virtual consultation, a patient suffered an injury (e.g. bang on their head) or a fall (e.g. mechanical or a medical event like syncope) and 158 (54%) reported they felt the current technological solutions are secure in terms of patient data. Qualitative data provided additional context to support the quantitative findings such as validity of indemnification, accuracy of diagnosis and consent using VC, safeguarding issues; and security and sharing of data. Potential changes to practice have been proposed to address these issues.

**Conclusions:**

VC have been rapidly deployed since the onset of the COVID-19 pandemic often without clear guidance or consensus on many important issues. This study identified legal, safeguarding, safety and security issues. There is an urgent need to address these and develop local and national guidance and frameworks to facilitate ongoing safe virtual orthopaedic practice beyond the COVID-19 pandemic.

**Supplementary Information:**

The online version contains supplementary material available at 10.1186/s12891-021-04113-y.

## Introduction

The outbreak of the 2019 novel coronavirus (COVID 19) was first reported in Wuhan, China and reached the United Kingdom on the 31st January 2020. The COVID-19 virus spreads primarily through droplets of saliva or discharge from the nose when an infected person coughs or sneezes. Social distancing measures have been established; the UK public were placed on ‘lockdown’ from the 23rd March 2020 [1] to avoid transmission of the disease.

Around a fifth of primary care consultations are for musculoskeletal problems [2, 3]. Consultations for patients with musculoskeletal problems may include a physical examination [4] [5], radiographs [4], exercises [6], self management [7], and manual therapy [8] (such as manipulation [9] or mobilisations [10]). Patients may have expectations of a thorough physical examination [11], individualised exercises, manual handling and ‘hands on’ treatment [12]. Virtual orthopaedic surgical [13–15] and rehabilitation [16–22] consultations have been found to be a viable alternative to face-to-face consultations. The outbreak of COVID-19 has led to a huge upsurge in the interest and importance of virtual consultations (VC) in practice [23–25] with patients being forced into undergoing VC. Much of the COVID-19 research around VC has focused on the process of rapid role out [23], its capability [26] and future direction [27, 28]. Some the research since COVID-19 has posed important questions around the issues of whether it is suitable for all [29], accessible for all [30] and how to overcome challenges to prevent medicolegal issues from occurring [31].

To our knowledge, no research has yet investigated issues encountered by orthopaedic and musculoskeletal clinicians using VC since the onset of COVID-19. The objective of the study was to explore orthopaedic and musculoskeletal clinicians’ views of potential legal, safety, safeguarding and security issues regarding the use of VC during the COVID-19 pandemic. A secondary objective was to suggest ways to overcome these issues.

## Methods

### Design

Mixed-method cross sectional survey.

### Ethics

This study was registered with the local Research and Innovation Centre. The Health Research Authority granted approvals on the 3rd August 2020 (IRAS ID: 244738).

### Questionnaire development

A questionnaire was developed by the study’s authors to explore issues encountered due to the use of VC during the COVID-19 pandemic. An initial consultation between the authors (AWG = research physiotherapist, GB and TB = specialist physiotherapists; AG = Consultant Orthopaedic Surgeon) identified four broad topics of importance. These topics were identified as important based on anecdotal experience of VC during the pandemic. The questionnaire was designed to quantitatively assess the presence of these phenomenon and qualitatively explore respondents’ views on these topics. The four topic areas of interest and definitions are shown below:

(i) Legal issues – which we define as those relating to indemnification between the patient and the clinician potentially resulting from their VC interaction.
(ii) Safeguarding issues – which we define as the process of protecting individuals for whom we care.
(iii) Safety issues- which we define as risks to the patient and clinician resulting from the VC interaction.
(iv) Security issues – which we define as risks resulting from data loss or data breaches because of the VC interaction.

Open textboxes were included to encourage participants to provide qualitative data. The questionnaire was piloted with five physiotherapists and five orthopaedic surgeons prior to dissemination of the final instrument to ensure that it was accessible to the target audience. No material changes were suggested to the questionnaire. The final questionnaire can be seen in the supplementary material (supplementary material 1).

### Participants and recruitment

The questionnaire was circulated via email and social media on the 4th August 2020 using the online platform ‘Survey Monkey’. The survey was disseminated across professional networks in the United Kingdom including the British Orthopaedic Association, the Association of Trauma and Orthopaedic Chartered Physiotherapists and the Digital Informatics Physiotherapy Group. The email encouraged colleagues to disseminate the survey widely. Participants were required to determine their eligibility prior to participating. Participants were eligible to participate providing they met the inclusion criteria shown in Table 1. This study sought to recruit at least 200 participants. This was a pragmatic decision that was made to ensure meaningful data analysis.

**Table 1:** Inclusion criteria

Inclusion Criteria	Exclusion Criteria
• Medically qualified professionals. OR • Practicing Allied Health Professional (eg physiotherapist, podiatrist). AND • Working in a trauma / orthopaedics / musculoskeletal setting. • Experience of conducting VC.	• No experience of conducting VC. • Unable to provide informed consent.

### Data analysis

Descriptive statistics were used to present quantitative data; inferential statistics were not used as this study sought only to identify the range of issues rather than make inferences from these data. A qualitative content analysis [32] was employed to identify empirical regularities within the data relating to these 4 themes outlined and defined above. We then mapped these to the coding framework which included the four issues of interest and ‘other issue’. This took the following forms:

i) A coding framework was developed consisting of the four issues outlined above and ‘other issue’
ii) Qualitative data were exported into an excel spreadsheet.
iii) Data were assigned a code by one author (AWG) to depict the type of issue represented within the data. The codes that were used related to the four issues of interest (legal issue, safeguarding issue, safety issue, security issue) or ‘other issue’ for those that did not fit within the definitions offered above.
iv) Codes were reviewed by a second author (GB). A third author (TB) was available to resolve any disagreements.
v) Data were organised into the coding framework based on the assigned code.
vi) A description of the type of content was presented for each issue.

### Reporting

This study was reported in accordance with the STROBE checklist for cross sectional studies and can be seen in the supplementary material (supplementary material 2).

## Results

### Responses

Three hundred and thirty-seven participants accessed the survey, 67 responses were empty and were therefore excluded, 310 responses were completed with a further 20 excluded as they were not using VC. Two hundred and ninety responses were included for analysis. Qualitative responses were coded relating to the four issues of interest. Of the 219 qualitative responses, 43 were coded as legal issues, 76 were coded as safeguarding issues, 79 were coded as safety issues, 10 were coded as security issues and 11 were coded as multiple / other. No disagreements between the first two authors arose in the allocation of coding. Quantitative data are shown in Table 2. Examples of qualitative data are shown in Table 3.

**Table 2:** Summary of quantitative results

**Question**	**Yes**	**No**	**Unsure**
Are you using virtual consultations with patients? (*n* = 298)	279 (94%)	19 (6%)	0 (0%)
Were you using virtual consultations before COVID-19 (*n* = 298)	30 (10%)	268 (90%)	0 (0%)
Do you feel the current technological solutions are secure in terms of patient data? (*n* = 296)	159 (54%)	24 (8%)	113 (38%)
Have you considered how, in a virtual consultation, you would handle an issue of safeguarding? (*n* = 298)	138 (46%)	38 (13%)	122 (41%)
Have you considered what you would do if, during a virtual consultation, a patient suffered an injury (eg bang on their head) or a fall (even if a medical event like syncope)? (*n* = 298)	130 (44%)	123 (41%)	45 (15%)
Does your professional indemnity cover you for any injuries sustained by patients during a virtual consultation? (*n* = 298)	44 (15%)	4 (1%)	250 (84%)
Have you encountered any other legal issues surrounding the use of virtual consultations since COVID-19? (*n* = 298)	258 (87%)	40 (13%)	0 (0%)

n = number of respondents

**Table 3:** Examples of qualitative data

**Theme**	**Qualitative Data**
Legal Issues *relating to indemnification between the patient and the clinician potentially resulting from their VC interaction*	• *Things relating to professional indemnity are vague … I am not aware of standards of clinical practice for virtual consultation which are the necessary starting point for defining the limits of indemnity insurance.* • *Remote consultation has been introduced without any universal standards so we are depending on the former system of protection to function within a very different context, and without any testing of the system to highlight weaknesses. I feel concerned that legally the person reporting an issue might be opening themselves up to litigation because the existing safeguarding legal framework might not apply to new ways of working.* • *I would value a legal opinion.* • *If working at home need to check with house insurance and council tax is covered* • *We have had discussions around what the outcome might be if a patient was recording the consultation without our knowledge and then attempted to use the recording as evidence for a complaint/legal case.* • *Would anticipate organisational cover as they have insisted we work in this way.* • *At the moment it is ok as its emergency pandemic setting, but that will all change when its being reviewed like all investigation’s years away from now and any guidance you can produce will help inform the future developments in this field.* • *Additionally, there is the massive area of effectiveness, a patient might try to pursue a negligence claim on the basis that the clinician was ineffective and missed something through using remote consultation. The whole area of remote diagnosis is litigation just waiting to happen*
Safeguarding Issues *the process of protecting individuals for whom we care*	• *I had a child who had injured themselves and I was concerned it was self-harm. I felt the consultation was difficult because the mother was with her in the room. She was 14. i was afraid to ask the mother to leave the room because I do not think I am allowed to be on the call with a child, it’s a very difficult situation. I had to involve the GP which I felt uncomfortable about.* • *Unsure if strictly legal but a young female patient was inappropriately dressed, nothing untoward was visible but I felt quite uncomfortable with the situation, had it happened in a F2F consultation I would have asked her to put clothes back on but the consultation appeared to have been in her bedroom and I wasn’t aware of her clothing until it was a bit too late.* • *I do not always see the child the consultation is about. So, I neither see nor talk to the patient.* • *We became aware post consultation that the patient’s brother was acting as the patient.* • *Feel concern that the NHS system surrounding safeguarding will not adequately support the new ways of working. I have not had any direct guidance about how things might need to adapt or if a possible safeguarding issue discovered through remote consultation requires different processes or different standards of evidence collection. Currently it feels as if the frontline clinician might be blamed for any failings in the system when in reality, frontline staff are working without a clear framework.* • *Follow usual policy, flag up with the safeguarding team.* • *Bring the patient in for an urgent face to face appointment.* • *If required contact the patients GP. If serious concerns, contact the emergency services as appropriate.*
Safety Issues *risks to the patient and clinician resulting from the VC interaction*	• *Assess the situation, if considered an emergency would call 999. If considered non urgent would give patient the appropriate advice eg to contact their GP or provide safety netting advice. Would check back on patient again later that day or ask patient to contact me. Would also document incident, complete clinical incident procedure, ring GP or write letter to GP (depending on nature of incident). This is currently not included in our SOP.* • *I am concerned that I am unable to fully risk assess the patient’s ability and environment so stick with safe practices which may not be the progression that is required.* • *If the patient were unresponsive on the other side of the camera, I would call 999. If the patient was responsive, I would discuss with them the best way forward (them phoning for assistance, me arranging assistance).* • *Patient is in their own home environment, so they are totally responsible. In fact, it should be considered as lucky episode as I will call 999 if I am worried about patient’s wellbeing with name and address* etc. *We cannot stop usefulness of virtual consultations. We must embrace it.*
Security Issues *risks resulting from data loss or data breaches because of the VC interaction*	• *Consider security of virtual platforms.* • *If I am being recorded [what if] that is then edited and or shared.* • *Data protection relating to setting used for consultation and privacy.* • *Data protection, consent, confidentiality, and risk.* • *Confidentiality, I do not know who else is either in ear shot or just off screen*. • *Patients taking calls in places that were not confidential or with unnamed people chipping in with inappropriate answers.* • *Data breach* • *Thank you for reviewing this area. I think we are all deeply involved in an area we just do not understand. At the moment it is ok as its emergency pandemic setting, but that will all change when its being reviewed like all investigation’s years away from now and any guidance you can produce will help inform the future developments in this field.*
Other issues *Issues identified that do not fit the above categories, or they fit within multiple categories*	• *Starting appointment on time has been an issue. The patient is sometimes busy with other work.* • *Deciding which platform to use is not straightforward and the Trust decision makers I have no doubt will not necessarily have the patients best interest at heart.* • *Students using virtual platform, adequate supervision for them.* • *Connection dropping, poor picture. Difficult to get a good angle to see patient properly. Patients can struggle to understand instructions without hand’s on feedback.* • *I think your examples are quite concerning as I have not really considered any of these issues. Advice and examples of all these issues are very much needed. Thank you for reviewing this area. I think we are all deeply involved in an area we just don’t understand. At the moment it is ok as its emergency pandemic setting, but that will all change when its being reviewed like all investigation’s years away from now and any guidance you can produce will help inform the future developments in this field. Thank you.* • *Remote form filling for investigations can be a challenge.*

### VC usage and demographics of participants

Of the 290 participants, 260 (90%) were not using VC prior to the COVID-19 pandemic, 206 participants (71%) were physiotherapists, 78 participants (27%) were medically qualified professionals, 6 were ‘other’ Allied Health Professionals (2%).

### Legal issues

Two hundred and forty-eight respondents (86%) were unsure whether their professional indemnity insurance covered VC. Just 42 respondents (14%) reported knowing that their indemnity covered them for injuries sustained during a VC. Some respondents assumed that this would be covered by their NHS Trust or that the indemnity would stretch to VC as they were operating within the scope of their public role. Others stated that since insurance providers allowed clinicians to undertake VC during the COVID-19 ‘lockdown’ they assumed that these may be covered moving forwards, but they were uncertain. One respondent questioned whether their home insurance covered them for conducting clinical consultations from home.

Participants reported the challenges of virtual diagnosis that would normally warrant a physical examination as well as the difficulties in delivering effective interventions which often require a hands-on approach. One respondent raised concerns about the effectiveness of a digital intervention:

*‘Additionally, there is the massive area of effectiveness, a patient might try to pursue a negligence claim on the basis that the clinician was ineffective and missed something through using remote consultation. The whole area of remote diagnosis is litigation just waiting to happen”.*

VC revealed potential issues of setting with examples including patients driving during a consultation, taking a call in a noisy and crowded environment and whilst cleaning a fishpond.

### Safeguarding issues

One hundred and thirty-six participants (47%) had considered how they would handle an issue of safeguarding whilst the remainder had not. Over half (56%) of participants had not considered or were unsure of safeguarding issues. Seventy-one participants commented, and strategies to handle safeguarding issues were suggested (see Table 3).

General themes emerged such that certain clinicians felt discouraged from using VC due to concerns over safeguarding such as a lack of visual clues on the area not within the view of the camera. One clinician reported being concerned with the potential of a patient having self-harmed and the challenges of being unable to conduct a formal assessment. One respondent reported a family member of the patient visibly naked in the background. One male clinician reported feeling uncomfortable at not being able to offer a chaperone to a female patient, whilst another reported feeling uncomfortable and vulnerable undertaking a VC with an inappropriately dressed young female patient.

It was noted by several participants that it was important to confirm the location of the patient at the outset of the consultation and to ask sensitising questions to identify issues of safeguarding. Some clinicians stated they would refuse to use VC for new patients purely due to the potential safeguarding risks.

Confidentiality was a frequent concern. It was difficult for clinicians to control who was present in the patient’s room and some reported situations where family members were answering on behalf of the patient and providing inappropriate answers. An example was given where a relative was pretending to be a patient. Several respondents reported concerns about being recorded.

### Safety issues

One hundred and twenty-six participants (43%) had considered what they would do if, during a virtual consultation, a patient suffered an injury (e.g. bang on their head) or a fall (e.g. mechanical or a medical event like syncope). More than half (57%) however, had not considered how they would respond if such a situation were to occur. Some participants reported that they had developed standard operating procedures to guide clinicians in what to do if such an event were to occur. A concern was raised about the logistics of managing students and the challenges of providing supervision to support their safe practice. Most respondents stated that they would call the emergency services or the patient’s general practitioner. It was deemed important to ascertain if the patient was alone at the start of the session and confirm the next of kin’s contact details.

### Security issues

Of the 290 participants, 158 (54%) reported they felt the current technological solutions are secure in terms of patient data. Twenty-three (8%) did not feel that the current technological solution is secure and 109 (38%) were not sure. Issues raised included security by the host platform (of data loss and third party recording); the recording of consultations by patients and subsequent sharing; and compliance with data laws such as General Data Protection Requirements (GDPR).

### Other issues

In addition to the four specific issues we investigated, we gained insights into a small number of other issues that were reported amongst the participant responses. These can be seen in Table 3.

## Discussion

Our study has identified a range of issues experienced by clinicians in a musculoskeletal setting, 90% of whom had not been using VC prior to the onset of the COVID-19 pandemic.

The issues have been categorised into legal (86%) such as ability to make an accurate diagnosis and validity of indemnification; safeguarding issues, such as the lacking of visual clues outside of the camera view; safety issues, such as how to deal with a patient falling in their home during a consultation; and security issues, such as data loss or breach of data laws.

### Legal issues

To indemnify is to ‘to pay somebody an amount of money because of the damage or loss that they have suffered’ [33]. All doctors and physiotherapists must have appropriate indemnity as a condition of their registration with the General Medical Council [34] and Health and Care Professions Council [35] respectively. Despite being registered with indemnity providers, 86% of respondents were unclear whether their indemnifiers provided cover for VC. It is the responsibility of the healthcare professional to ensure they have adequate coverage both for patient and public liability.

Another concern was raised in relation to professional versus public liability. Public liability insurance is usually taken by a hospital or clinic to protect individuals who come to harm in or on the insured property. However, when it comes to VC, the patient is usually in their own property which may not be covered.

Accuracy of diagnosis and adequacy of consent using VC themes were raised. Delays in diagnosis features as the most common cause for litigation in hip fracture claims [36]. Wade and colleagues [37] identified VC as a potential cause for reduced diagnostic accuracy. This might lead to increased liability due to lower quality consultations. In a review of successful litigation against English Health Trusts in the treatment of adults with orthopaedic pathology [38] seventy-eight cases of “poor consent process” resulted in successful litigation claims. The use of VC during the consenting process may impact on the discussion of treatment options and risks.

### Safeguarding issues

Self-cutting has previously been identified as the most common form of deliberate self-harm representing 45% of self-harm cases [39]. A large proportion of respondents had not considered safeguarding issues (53%) and participants comments identifies concerns in this area due to a combination of technology restraints, lack of external clues, and privacy. VC can adversely affect the flow of the conversation [40] which may impact a clinicians attempt to undertake a thorough subjective assessment. Poor internet connection is commonly reported [41] and interferes with transmission of information [42].

Lack of confidentiality may manifest through a lack of physical privacy if the patient’s environment is not conducive to having a quiet space for their appointment [37]. Cranen and colleagues [43] found that patients suffering with chronic pain valued telerehabilitation but hesitated to use it as an autonomous treatment. Patients expressed concern at feeling alienated through telerehabilitation and service providers may feel inclined to offer virtual groups to facilitate fellow sufferer contact which may also lead to confidentiality issues.

### Safety issues

Protocols for managing the fallen patient exist within a healthcare setting [44]. Virtual rehabilitation may include strengthening and balance exercises designed to challenge patients’ proprioception, particularly those susceptible to falls. Less than half of respondents in our study had considered safety issues such as falls and how to manage them in VC and guidance published in response to COVID-19 does not appear to consider this either [26].

A study of telephone consultations in primary care found that clinicians expressed strong concerns about safety being compromised as a result of lack of formal and informal examination [45]. Guidance to ensure the safety of patients and clinicians in delivering VC is needed.

### Security issues

Concerns about data security are not new [37] and were raised by participants in our study. In the UK, NHSX is a joint unit bringing together teams from the Department of Health and Social Care and NHS England and NHS Improvement to drive the digital transformation of care. NHSX released guidance for healthcare professionals during the pandemic [46] which included relaxation of governance restrictions to optimise virtual care. The Chartered Society of Physiotherapy (CSP) released guidance for physiotherapists around rapid implementation of remote consultations [47] which included (if no other alternative) using own devices and commercial apps where there is no practical alternative and the benefits outweigh the risks [46].

Healthcare data breaches account for three-quarters of overall data breaches in the last 5 years [48]. Of these, hacking and malicious attacks have affected over 145 million individuals between 2015 and 2019. A number of data breaches occurred in the first half of 2020 [49], with access of personal data featuring in all ten highlighted cases. A survey of 43 senior plastic surgeons [50] elicited their views on the future of virtual consultations in plastic surgery; 38% of respondents were not aware of encryption and 48% were unaware of GDPR compliance. As the number of remote consultation platforms grow, it is essential that patients and clinicians are aware of their potential security issues and that policies and procedures reflect increased risks [51].

In addition, the recording of VC either by the clinician or the patient and subsequent sharing, for example on social media [52], is an area of controversy which needs further research. A recent study [53] provided an overview of legal considerations for both patients and clinicians that focuses around consent, sharing of recordings, ownership of recordings and data security and storage. Eleven states in the USA require all party consent to legally record a conversation [52]. In contrast, in the UK, patients and relatives can record a consultation without the clinicians consent, because the information being recorded is personal to them and is exempt from the Data Protection Act (DPA) and General Data Protection Regulation (GDPR) [54].

### Strengths and limitations

This study must be considered in light of its limitations. The questionnaire was developed by the authors and as a result the phenomenon of interest investigated may have been subject to bias. A wider range of questions may have identified additional issues of interest. Although no disagreements arose in coding of qualitative data, this may be due to the general definitions decided upon at the outset of the study. Recruitment of a more diverse range of participants, a higher volume of participants and engagement with pre-existing theory or frameworks may have led to more generalisable results. Reporting bias may be present if respondents with negative views favoured participation. As with all surveys, recall bias may occur. That said, this pragmatic study is believed to be the largest survey of musculoskeletal clinicians to date to investigate issues of VC due to COVID-19 and it offers a potential starting point for discussion across the orthopaedic and musculoskeletal community. A strength of this study is that it was developed and shared during the pandemic; the questions were developed by clinicians experienced in developing and enacting virtual consultation pathways due to COVID-19. The closed nature of questions within the questionnaire provided an indication of the prevalence of issues across our sample and the open questions provided useful qualitative data and insights that can be used to guide development and refinement of future policy to address issues identified. This study provides a potential starting point for future research to expand upon.

### Implications for future practice and clinical research

Our study identified several potential issues in clinical practice. Table 4 offers some potential suggestions for practice:

**Table 4:** Potential suggestions for practice

Issue	Suggestions
Legal	(i) Provide information for patients about what a VC can and cannot do. (ii) Establish a ‘code of conduct’ that provides patients with information about what is acceptable. (iii) Ensure adequate indemnity insurance (including Cybercover) is in place for the individual and organisation and that policy exclusions are transparent.
Safeguarding	(i) Establish a robust process of patient identification. (ii) Ensure patient has access to a chaperone. (iii) Ensure safeguarding policies at institution consider issues of VC.
Safety	(i) Provide guidance for patients on establishing a safe environment for virtual consultations. (ii) Conduct risk assessment of physical environment patient-side. (iii) Establish protocols for clinical assessments. (iv) Establish procedures for managing patient incidents during virtual consultations.
Security	(i) Establish clear guidance on the security of the various technology platforms available. (ii) Determine whether data is encrypted (iii) Establish that appropriate approvals are in place by providers and indemnifiers. (iv) Complete Data Protection Impact Assessment prior to roll out, considering relevant data protection guidance and policy.

The COVID-19 pandemic has changed the virtual healthcare landscape. Pragmatic evaluation of virtual pathways would assist in the identification of additional issues not alerted in this work. Thorough evaluation of patient and clinician experience, in addition to service outcomes, is essential to determine effectiveness and acceptability.

## Conclusion

VC have been rapidly deployed since the onset of the COVID-19 pandemic often without clear guidance or consensus on many important issues. This research has identified legal, safeguarding, safety and security issues relating to VC for musculoskeletal care during the COVID-19 pandemic. During the first wave of the pandemic, emergency rules applied, and many participants within this study had not considered many of the issues explored in this study. Careful consideration must go to whether research published prior to COVID-19 serves as an appropriate basis for post COVID-19 policy. Future policy should be developed following a thorough appraisal of the safety and effectiveness of VC employed since COVID-19. As we move towards the next waves of the pandemic, there is an urgent need to address these issues and plug the gaps. This study has highlighted how some clinicians across musculoskeletal and orthopaedic care may not have an awareness of these concerns. Focusing on potential legal, safeguarding, safety and security issues and proactively addressing them may facilitate ongoing safe virtual orthopaedic practice beyond the COVID-19 pandemic.

## Supplementary Information

**Additional file 1.**

**Additional file 2.**

**Additional file 3.**

## Data Availability

Data from this study are available upon reasonable request to the corresponding author.
