# Supplementary material for: A mixed-methods survey to explore issues with virtual consultations for musculoskeletal care during the COVID-19 pandemic
Source: BMC Musculoskelet Disord. 2021 Mar 5;22:245. doi: 10.1186/s12891-021-04113-y (PMC7933396; doi:10.1186/s12891-021-04113-y)
Supplement: Supplementary file 1 — Additional file 1. [file 12891_2021_4113_MOESM1_ESM.pdf]

Participant Information Sheet

We are carrying out a study to assess the impact that COVID-19 has had on the use of virtual consultations and would be grateful if you could answer some questions. This survey will take less than 5 minutes to complete.

This study has been approved by the Health Research Authority (IRAS ID: 244738) and is sponsored by the Royal National Orthopaedic Hospital.

The Participant Information Sheet is available by clicking this link (opens in a new window).
So that we can be sure you understand the study and would like to take part, please read the following statements and tick the box if you understand and agree with them. Please feel free to contact a member of the research team if you have any questions.

1. Please select the following statements if you agree with them:

- [ ] I have read and understood the information sheet (version 2, dated 3rd August 2020) and agree to take part in this study and my data progressed accordingly
- [ ] I certify I am a practicing clinician working in musculoskeletal or orthopaedic care

2. Are you using virtual consultations with patients?

- Yes
- No

3. Were you using virtual consultations before COVID-19

- Yes
- No

4. Do you feel the current technological solutions are secure in terms of patient data?

- Yes
- No
- Not Sure

5. Have you considered how, in a virtual consultation, you would handle an issue of safeguarding?

- Yes
- No
- Not Considered

Comments

6. Have you considered what you would do if, during a virtual consultation, a patient suffered an injury (eg bang on their head) or a fall (even if a medical event like syncope)?

- Yes
- No
- Not Sure

Comments

7. Does your professional indemnity cover you for any injuries sustained by patients during a virtual consultation?

- Yes
- No
- Not Sure

Comments

8. Have you encountered any other legal issues surrounding the use of virtual consultations since COVID-19?

- No
- Yes - please specify

9. What is your professional background?

10. Please leave your email address if you would like to be contacted about this or further research, or receive the results of his survey
