# Supplementary material for: A mixed-methods survey to explore issues with virtual consultations for musculoskeletal care during the COVID-19 pandemic
Source: BMC Musculoskelet Disord. 2021 Mar 5;22:245. doi: 10.1186/s12891-021-04113-y (PMC7933396; doi:10.1186/s12891-021-04113-y)
Supplement: Supplementary file 3 — Additional file 3. [file 12891_2021_4113_MOESM3_ESM.docx]

# Title Page

# A mixed-methods survey to explore issues with virtual consultations for musculoskeletal care during the COVID-19 pandemic

Manuscript Author List:

1. Anthony Gilbert. Therapies Department, Royal National Orthopaedic Hospital & University of Southampton, School of Health Sciences
2. Gregory Booth. Therapies Department, Royal National Orthopaedic Hospital
3. Tony Betts. Therapies Department, Royal National Orthopaedic Hospital
4. Andy Goldberg. UCL Institute of Orthopaedics and Musculoskeletal Science, University College London & Trauma and Orthopaedics Department, Wellington Hospital

Corresponding Author: Anthony Gilbert. [anthony.gilbert@nhs.net](mailto:anthony.gilbert@nhs.net)

Tel: +442089095820

Postal Address:

Anthony Gilbert

Therapies Department

Royal National Hospital

Stanmore

Middlesex

HA7 4LP
